# Nursing guidelines for caregivers of children with congenital heart disease after discharge: Integrative Review

**DOI:** 10.17533/udea.iee.v41n3e05

**Published:** 2023-10-21

**Authors:** Bruna Alves Machado Amazonas, Denise Maria Guerreiro Vieira da Silva, Maria de Nazaré de Souza Ribeiro

**Affiliations:** 1 Registered Nurse, MSc. Email: brunaalvesmachado37@gmail.com brunaalvesmachado37@gmail.com; 2 Registered Nurse, PhD, Full Professor, Universidad del Estado del Amazonas, Amazonas-Brasil. E-mail: denise_guerreiro@hotmail.com Universidade do Estado do Amazonas Universidad del Estado del Amazonas Amazonas Brazil denise_guerreiro@hotmail.com; 3 Registered Nurse, PhD, Full Professor, Universidad del Estado del Amazonas, Amazonas-Brasil. E-mail: mnribeiro2@gmail.com Universidade do Estado do Amazonas Universidad del Estado del Amazonas Amazonas Brazil mnribeiro2@gmail.com

**Keywords:** nursing care, nursing, heart defects, congenital, caregivers, children, atención de enfermería, enfermería, cardiopatías congénitas, cuidadores, niños, cuidados de enfermagem, enfermagem, cardiopatias congênitas, cuidadores, crianças

## Abstract

**Objective.:**

To identify the nursing guidelines for caregivers of children with congenital heart disease (CHD) after hospital discharge.

**Methods.:**

This is an integrative literature review of articles published between 2016 and 2022. In order to select the studies, the controlled descriptors “Nursing Care”, “Nursing”, “Heart Defects, Congenital”, “Caregivers” and “Child” were used in four scientific databases - LILACS, SCIELO, PUBMED and BDENF.

**Results.:**

The current integrative literature review analyzed 11 articles from the original sample. The main nursing care issues are those related to nutrition, oral health, leisure and physical activity, care with medication and the surgical wound, as well as the need to offer support to these children’s families. The authors emphasize that nurses are present at various moments in a child’s life, including at birth, but the approach to CHD is scarce in their basic training as nurses, as well as in their professional practice, and there is a shortage of continuing education proposals for the care of children with CHD.

**Conclusion.:**

The study showed that nursing guidelines are focused on basic care and family support for these children. Lastly, this study highlighted the important role of nurses in terms of consolidating guidelines on the care needs of these children.

## Resumen

**Objetivo.:**

Identificar las orientaciones de enfermería para los cuidadores de niños con cardiopatía congénita (CC) después del alta hospitalaria.

**Métodos.:**

Se trata de una revisión bibliográfica integradora de los artículos publicados entre 2016 a 2022. Se utilizaron los descriptores controlados “Nursing Care”, “Nursing”,“Heart Defects, Congenital”, “Caregivers” y “Child” para seleccionar los estudios en cuatro bases de datos científicas - LILACS, SCIELO, PUBMED y BDENF.

**Resultados.:**

Se analizaron 11 artículos de la muestra original en esta revisión. Los principales tipos de cuidados de enfermería son los aspectos relacionados con la nutrición, la salud bucodental, el ocio y la actividad física, los cuidados con la medicación y la herida quirúrgica, así como la necesidad de ofrecer apoyo a las familias de estos niños. Los autores enfatizan que, aunque, las enfermeras están presentes en diversos momentos de la vida del niño, incluyendo el nacimiento, el abordaje de la ECC es escaso en su formación básica como enfermeras, así como en su práctica profesional, y faltan propuestas de formación continua en el cuidado de estos niños.

**Conclusión.:**

El estudio mostró que las orientaciones de enfermería se centran en los cuidados básicos y en el apoyo familiar a niños con ECC. Por último, este estudio se evidenció el importante papel de las enfermeras en la consolidación de las orientaciones para las necesidades de atención de estos niños.

## Resumo

**Objetivo.:**

Identificar as orientações de enfermagem para cuidadores de crianças com cardiopatias congênitas (CC) após alta hospitalar.

**Métodos.:**

Trata-se de uma revisão integrativa da literatura de artigos publicados entre 2016 e 2022. Para seleção dos estudos, foram utilizados os descritores controlados “Nursing Care”, “Nursing”, “Heart Defects, Congenital”, “Caregivers”), y “Child” em duas bases de dados científicos - LILACS, SCIELO, PUBMED e BDENF.

**Resultados.:**

Na presente revisão integrativa da literatura, foram analisados onze artigos da amostra original. Os principais cuidados de enfermagem são os relacionados com à alimentação, saúde bucal, lazer e atividade física, cuidados com medicamentos e com a ferida operatória, como também a necessidade de oferecer apoio à família dessas crianças. Os autores ressaltam que o enfermeiro está presente em diversos momentos da vida da criança, inclusive, no nascimento, porém a abordagem sobre CC é escassa na sua formação básica como enfermeiro, assim como em sua atuação profissional, percebendo-se escassez de propostas de educação continuada para o cuidado de crianças com CC.

**Conclusão.:**

O estudo apontou que as orientações de enfermagem estão voltadas ao cuidados básicos da assistência e ao apoio familiar dessas crianças. Por fim, foi evidenciado nessa pesquisa o importante papel do enfermeiro para a consolidação das orientações das necessidades voltadas ao cuidar dessas crianças.

## Introduction

Congenital heart defects (CHD) are abnormalities that affect the heart and great vessels, which are responsible for important functions in live births and are more common in fetuses. CHD can be clinically divided into cyanotic and acyanotic, which indicate the presence or absence of a bluish coloration of the skin and mucous membranes due to a deficit of oxygen in the blood.^1^^,^^2^ Among the various existing cardiac malformations, CHD have an impact on children’s lives, on morbidity and mortality and on the increase of expenditure in public and private health services.^3^ They are one of the biggest causes of death among cardiac malformations in the first year of life, accounting for around 2 to 3% of neonatal deaths and are an important cause of mortality.^4^

Emerging countries, where access to health care is more difficult, tend to have higher mortality rates than developed countries, according to national epidemiological studies.^5^ Approximately 36,000 children are born with CHD in the United States of America (USA) each year, corresponding to 1% of all live births. Of this percentage, 44.5% die in their first year of life.^6^ In Brazil, CHD remains the third leading cause of death in the neonatal period. The Brazilian Ministry of Health estimates that the incidence in the country is 28,846 new cases of CHD a year, but notifications by the Unified Health System (SUS, as per its Portuguese acronym) show that there are as many as 1,680 cases a year, demonstrating flaws in the diagnosis and identification of the problem.^7^ Nevertheless, around 23,000 children need surgical treatment in the first year of life, while only 6,000 are operated on. In the North and Northeast regions, this rate is quite peculiar, as up to 80% of children with CHD are not diagnosed and do not undergo treatment.^8^

Genetic factors are associated with the pathogenesis and appearance of various cardiac malformations and around 400 genes are involved in congenital heart disease.^9^ Nevertheless, CHD is related to various forms and a variety of causes, such as extrinsic factors, which can directly or indirectly affect the development of the fetus at the embryonic stage. These factors are related to the use of thalidomide, retinoic acid, alcohol use by the mother, hypoxia, other medications and hypovitaminosis. During pregnancy, intrinsic maternal factors can influence the development of CHD, such as gestational diabetes, maternal obesity, phenylketonuria, viral infection and hyperthermia.^10^^,^^11^ In underdeveloped countries, access to health care is precarious and reveals difficulties in different aspects, such as poverty, insecurity, housing issues, education and family understanding of the disease, immigration, access to food and barriers in terms of moving around and transportation. All these factors contribute to the clinical outcomes of people suffering from cardiovascular diseases, whether they are adults or children.^12^

Congenital heart disease has an impact not only on children’s lives, but also on those of their caregivers and family members. The range of recurrent invasive treatments, surgeries and increased risk of death lead to the development of stress and damage to mental health. Parents of children with CHD tend to have less time for leisure, problems in terms of keeping a job and work overload, resulting in social isolation and family financial problems.^12^ Comprehensive care for children with congenital heart disease has been established since 2017, through a federal project by the Brazilian Ministry of Health. The project aims at expanding care for children with CHD, increasing care for these children by 30% per year, with more than 3,400 procedures, totaling around 12,600 procedures per year, with a direct impact on the reduction of neonatal mortality.^13^

Given the evolution of the diagnosis and treatment of CHD, there is no need for just one professional with specialized training, but rather the work of different professionals with different training and specialties to work together in a complementary, integrated and simultaneous way to care for children with this condition. These professionals must develop an active partnership with the public health system, resulting in more team training, in order to guarantee early diagnosis and appropriate treatment.^14^^,^^15^ Nurses are present at various moments in a child’s life, including at birth, but the approach to CHD is scarce in their basic training as nurses, as well as in their professional practice, and there is a shortage of continuing education proposals for the care of children with CHD. Professionals caring for neonates with CHD need to be prepared in a systematic and continuous way, through their participation in the teaching-learning process and in health education with family members.^16^^,^^17^ However, even if the nurse’s approach takes into account the systematization of nursing care (SNC), specific knowledge about nursing care for children with CHD must be continuously improved. In addition, family caregivers of children with CHD need to better understand the disease and develop skills to help with home care after hospital discharge. The objective of this study was therefore to identify the nursing guidelines for caregivers of children with congenital heart disease after hospital discharge. 

## Methods

This is an integrative literature review (ILR) through an exploratory study. The present research was developed in six moments: 1 - identification of the problem to be solved and elaboration of the research question; 2 - development of inclusion and exclusion criteria for articles; 3 - extraction of relevant information from the selected studies; 4 - evaluation of the studies selected to compose the integrative review; 5 - reading and interpretation of studies; 6 - organization, discussion and complete synthesis of publications.^18^ In the first stage, the PICO strategy was used to formulate the guiding question, being P (population), I (intervention), CO (context).^19^ Therefore, the following question was elaborated: “(P) For caregivers of children with congenital heart disease (I) What are the nursing guidelines after hospital discharge? (Co) Not applicable.”

In the second stage, the search was carried out between April and July 2022. As for the inclusion criteria, articles were selected that covered the topic and were aimed at the study’s objective, available in full in electronic form free of charge, in Portuguese, English and Spanish; published between 2016 and June 2022, considering this time necessary for the quality of this study’s proposal, since the temporality of six years of search allows its product to present a current discussion on what was investigated. Exclusion criteria were articles that did not address the relevant theme for the scope of the research; were not electronically complete; written outside the delimited period, as well as editorials, publications in event proceedings, theses, dissertations, monographs and incomplete documents; and were not available in full online. 

For the search strategy, the following descriptors established in *Descritores em Ciências da Saúde* (DeCS) (DeCS) and Medical Subject Headings (MeSH) were used: “Nursing Care”; “Nursing” (“Nursing”); “Congenital Heart Defects” (“Heart Defects, Congenital”); “Caregivers” (“Caregivers”); “Child” (“Child”), related to the Boolean operators AND and OR. For data selection, articles published in scientific journals were analyzed, using the databases of the Virtual Health Library Portal (VHL), such as: Latin American and Caribbean Health Sciences Literature (LILACS), PUBMED and *Base de Dados de Enfermagem* (BDENF). For the search in the Scientific Electronic Library Online (SCIELO), the page “scielo.br” was used. The used databases were selected because they are a reference in the health and nursing areas. 

**Figure 1:**
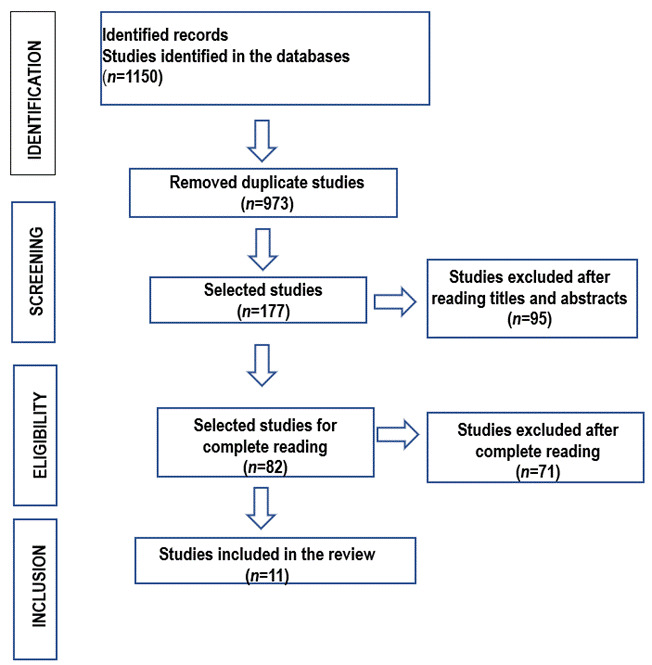
Flowchart of the study selection process

In order to ensure the quality and reliability of the study, the recommendation Guideline PRISMA (Preferred Reporting Items for Systematic Reviews and Meta-Analyses) was adopted, which is composed of a structured checklist, capable of describing all the important and essential steps and approaches for the preparation of a review, added to the flowchart (FIGURE 1) that discriminates the elements of the methodology for identification, selection, eligibility and inclusion of references.^20^^,^^21^ Several classifications are available in the literature, but the most classic score for classifying primary studies was systematized by the Oxford Centre for Evidence-Based Medicine (CEBM) in 1998, and its last update was carried out in 2009.^22^

A total of 1,150 publications were identified, of which 973 were excluded after applying the filters and inclusion and exclusion criteria, leaving 177 articles. Subsequently, the selection was carried out by reading the articles considered potentially eligible in their entirety, whose titles and abstracts provided evidence on the subject, where 71 articles were excluded and 11 articles were included in the review (Figure 1). The selection steps were carried out. For the storage, transcription and analysis of the articles, a protocol was elaborated by the authors. After defining the sample, a database was created using Microsoft Excel® 2016 software, which allowed organizing and compiling information from the selected studies. The presentation of the results and their discussion were carried out in a descriptive way, allowing the reader to evaluate the applicability of the literature review, in order to positively impact nursing practice, providing an organized way to review the evidence on a topic. The level of evidence of the selected studies was based on the classification that categorizes research into seven levels of evidence, where “1” is the level of greatest evidence: I- systematic reviews with meta-analysis of clinical trials; II- randomized clinical trial; III- clinical trial without randomization; IV- cohort, case-control; V- systematic review of descriptive works; VI- single descriptive or qualitative study; VII- opinion of expert committees or authorities.^21^

## Results

In this integrative literature review, 11 articles from the original sample were analyzed. For a better organization of the selected studies, information such as author, year, title, database, origin of the study and journal of publication were extracted, thus facilitating the process of interpretation of the obtained results and of the articles selected for the composition of the current study. The articles were predominantly published in the years 2020 (*n*=2) and 2019 (*n*=5), with most publications in Portuguese (*n*=6). Of the 11 studies, six were conducted in Brazil and five in the United States. The journals that most contributed were those in the nursing area (*n*=7), followed by the medical area (*n*=4). Accordingly, the included studies were found in BDENF, four in SCIELO, five in PUBMED and one in LILACS. (Table 1)

**Table 1:** Descriptive variables and levels of evidence of the 11 articles included in the integrative review.

Article	Authors, year; country (reference)	Study design	Objective	Results and Conclusions/Outcomes	Level of evidence
A1	Precce *et al*., 2020, Brazil.^23^	Qualitative	To analyze the educational demands of family members of children with special health needs in the transition from hospital to home.	The information was analyzed and synthesized into three thematic categories Recognizing congenital heart disease; Caring for the neonate in the intensive care unit; the family and the care for the neonate. There is a need for greater professional involvement in terms of caring for these children and there are gaps in nurses’ knowledge production that show this reality, in such a way as to subsidize evidence-based clinical practice.	4
A2	Xavier *et al.*, 2020, Brazil.^24^	Exploratoryand descriptive research with a qualitative approach	To understand the meanings attributed by family caregivers to the diagnosis of chronic illness in a child.	The family attributed meanings to discovering the chronic illness diagnosis in a child when interacting with nursing/health professionals. The family’s interaction with the nursing/health team contributes to the meaning attributed by the family member to the chronic illness diagnosis in a child.	4
A3	Witkowskia *et al*., 2019, Brazil.^25^	Descriptive and cross-sectional study	To present the experience of training family members of children and adolescents participating in a multi-professional program at home.	This study presented the results of training family caregivers of children and adolescents in home care. In conclusion, it showed that the process of dehospitalizing children and adolescents can be viable, safe and effective through training.	4
A4	Flerida *et al*., 2019, USA.^26^	Exploratoryand descriptive research with a qualitative approach	To describe the perceptions and experiences of mothers of babies discharged from hospital after surgery for complex congenital heart disease.	The analyses led to the development of one category, “having to be the only one”, which had 3 properties: having no choice but to provide complex care at home, dealing with unexpected roles and dealing with the possibility of death. To highlight the experiences of mothers who provide medicalized care at home for their babies after complex heart surgery. The role of the caregiver is vital, but challenging. Mothers’ care at home can be improved by nursing interventions, such as routine screening, as well as evaluation of changes in family coping.	4
A5	Mirjam *et al*., 2019, USA.^27^	Qualitative, descriptive and exploratory study	To explore the experiences of mothers and fathers from the prenatal or postnatal diagnosis of the congenital heart disease of the newborn to the first discharge after heart surgery.	Between the diagnosis and discharge of the child from hospital after heart surgery, the main theme for parents was coping with demanding emotional and practical work. Health professionals must establish trusting relationships with parents while continuously accompanying families, providing consistent and direct information and expressing appreciation for the exceptional emotional and practical work of parents. The awareness of health professionals in relation to the experiences of parents is vital for compassionate family-centered care.	4
A6	Janie *et al*., 2019, USA.^28^	Qualitative, descriptive and exploratory study	To create and test a visual bedside tool to increase parents’ partnership with nursing in the development of supportive child care after cardiac surgery.	Practical staff training and informal bedside education in developmental care are needed to educate staff on how to support parents in terms of providing adequate physical care and developmental stimulation for their babies.	4
A7	Ju Yeon, *et al*., 2019, USA.^29^	Exploratoryand descriptive research with a qualitative approach	To investigate the needs of mothers to form partnerships with nurses during the postoperative recovery of children in a pediatric cardiac intensive care unit.	Mothers wanted information about the post-operative stability of their babies in the early stages of recovery and hospital discharge. The condition of the babies strongly influenced the needs of the mothers in relation to partnerships. Therefore, the nurse had to provide information to the mothers individually and encourage them to participate in the care.	4
A8	Alves JMNO, *et al*., 2017, Brazil.^30^	Qualitative, descriptive and exploratory study	To understand the experience of the care partnership by the parents of children with special health needs.	Partnership opportunities include empowering parents and decision-making in partnership, established in a dynamic, unique and continuous relational process. Partnership opportunities are a fundamental prerequisite for providing care that focuses on the child and their parents as resources.	4
A9	Elisabeth Bruce, *et al*., 2017, USA.^31^	Descriptive research with a qualitative approach	To shed light on the perceptions of pediatric nurses (PN) about supporting families with a child with a congenital heart defect.	The analysis revealed that the nurses feel that letting parents get involved in their child’s care is of great importance in terms of supporting families. Although they have a paternalistic attitude towards families, they also stated that nurses should inform parents about child care and create a good relationship with the family and build trust between all involved parties.	4
A10	Magalhães, *et al*., 2016, Brazil.^32^	Descriptive research with a qualitative approach	To analyze the educational demands of family members of children with special health needs in the transition from hospital to home.	The educational demands of family members of children with special health needs in the transition from hospital to home come from the clinical care of the child's body, and originate from complex and continuous care, technological care, modified habitual care, medication, developmental care and mixed care.	4
A11	Okido, *et al*., 2016, Brazil.^33^	Qualitative, descriptive and exploratory study	To understand the experience of mothers of technology-dependent children in relation to medication care.	The experiences of these mothers with medication care are permeated by daily challenges, including maternal overload and feelings of anxiety. It is also suggested that nursing develop family-centered care, acting as a facilitator in the process.	4

The concept of assistance has been gaining great repercussions and gaining notoriety with regard to the care of children with CHD. This care must be individualized, offering quality, comfort and safety. It is essential that the team provides adequate and complete assistance. The child with CHD has specificities, needs differentiated care aimed at maintaining cardiac function and its needs. Accordingly, another important aspect to be considered refers to the difficulties related to the complexity of the treatment and care required by the child’s illness. This care is so significant that it can change the family’s routine, directly affecting the caregiver’s personal and professional life. Some parents find it difficult to keep their jobs, which further restricts their financial resources, which are used only for the basic needs of the family and directed towards the treatment of the child.

The main nursing care procedures are described according to the studies selected for the composition of this ILR. In this context, it is noteworthy that, among the main care among the articles analyzed in this research, aspects related to food, oral health, leisure and physical activity, care with medication and the surgical wound, as well as the need to offer support to the family of these children, are highlighted. Such care is closely related to that offered by the nursing team during hospitalization, as well as after hospital discharge. By having direct access to the child and his/her caregiver, the nurse occupies an important place in this care, thus having greater opportunities to identify needs.

In this context, it is necessary for nursing professionals to guide family members about the essential care for these children with CHD, who, through their technical and scientific knowledge and the rescue of the essential humanization of care, provide assistance of quality aimed at the promotion, maintenance and recovery of health, observing the human being in its entirety, thus enabling well-being in the emotional, physical and psychological spheres.

It was found that 100% of the studies expressed Level of Evidence 4 and described outcomes that indicate the importance of the family in terms of attributing the meaning of the disease to the interaction with health professionals in such a way that care is effective. The articles were separated and composed two categories for discussion: care needs pointed out by family caregivers and educational demands that can be met by nurses.

## Discussion

### Care needs pointed out by family caregivers

Childhood hospitalization is characterized as a period of fear and uncertainty for children and their families, who need the help of nursing professionals, especially when it comes to coping with a chronic illness.^37^ In the A2 study,^24^ the contribution made by these professionals to participatory assistance focused on caring for children with chronic illnesses and their families, agreed in line with the child’s needs, can be observed. The family members suffer when they realize the incurable diagnosis in a child, mainly because they have difficulty in terms of dealing with this reality. Chronic childhood illnesses, because they are incurable, cause sequels over time, imposing limitations on the child, requiring special care skills and competencies from family caregivers for their rehabilitation, requiring training, supervision and observation of care. In this sense, it can be realized that the family, when interacting with the nursing team, seeks to share feelings and perceptions in the face of the finitude and fragility of the human condition that a chronic and severely serious illness imposes.^26^ The experiences of children with complex congenital heart disease after hospitalization and family care at home can be improved by nursing interventions, such as routine screening for infant distress, as well as evaluation of changes in family coping or relational challenges that threaten family function (study A4).^26^ According to the authors, the analysis led to the development of a perception that few professionals address the care versus the experiences of parents caring for babies with complex congenital heart disease who are discharged from hospital.

Also aiming for an approach based on a constructivist paradigm, study A5^27^ reports the need for health professionals to establish trusting relationships with parents, while continuously accompanying families, providing consistent and direct information and expressing appreciation for the exceptional emotional and practical work of parents. The awareness of health professionals in relation to the experiences of parents is vital for compassionate family-centered care. Study A7^29^ investigated the needs of mothers to form partnerships with nurses during the postoperative recovery of children in a pediatric cardiac intensive care unit. Nurses are seen as part of the multiprofessional team, facilitators of knowledge and with scientific training capable of collaborating positively in the process of promoting care for children with morbidities when they return home. In A10,^(32^ the study demonstrated the need for greater involvement by nurses to improve nursing care for these children, carefully emphasizing that there are still gaps in the production of knowledge by nurses that show this reality, in such a way as to subsidize evidence-based clinical practice. It should be considered that the path to improving care is always that of science, with the development of studies that will strengthen this care, unifying theory and practice is a dialogical integration for the construction of knowledge.^39^ The complex care required by these children, such as oxygen therapy, the use of a tracheostomy with or without mechanical ventilation, enteral feeding, dialysis and a continuous medication regimen, entail a heavy burden on caregivers at home.^36^

In this sense, study A11^33^ addresses the need for knowledge of the disease, the importance of treatment and mastery of care techniques, significantly reducing the anxiety and stress levels of these caregivers. Thus, according to the study, communication between the family and the health service is essential for building knowledge and empowering the caregiver. In view of the above, nursing becomes the protagonist of care for these children, thus contributing to the improvement and effectiveness of care provision. To this end, nursing care must ensure quality and safety in an individualized way for each person, enabling care based on scientific evidence.

### Educational demands that can be met by nurses

Study A1^23^ brings up the educational demands related to clinical care in this context, indicating that the work of nursing professionals must be closely related to preparing family caregivers for the development of care in hospital with a view to discharge. Nevertheless, discharge needs to be conceived in a procedural way, in order to include the planning and preparation of families. It is therefore up to nurses, in the health-disease transition, to mediate innovative, complex and continuous care, as well as equipping family members to care for, respecting their knowledge, encouraging reflection, action and empowerment.^35^ Study A3^25^ addresses the process in which, at the same time as the dedication of trained family members is needed to care for children and adolescents after hospital discharge, certain prerequisites are essential for the child to be able to go home safely, leading to successful rehabilitation. To share information about developmental support care provided by parents during each shift, thus generating opportunities for parents to initiate the care of their children, was the focus of study A6,^28^ which aimed at strengthening the importance of the bedside care partnership in terms of preparing parents, family well-being and child outcomes providing comfort, assisting in the daily care routine of these children.

In study A8,^30^ important nurse attitudes were identified, such as respect, trust, empathy and advocacy. These attitudes are described as attributes of the partnership model. Relationships established between parents and health professionals that meet these requirements are characterized as promoting parental empowerment. The adaptation on the part of parents to caring for their children at home requires a broad network of family and social support, with the close involvement of health professionals. Nursing care based on the philosophy of family-centered care and the care partnership model is considered ideal to help them to fulfill the role they will play.^34^

To illuminate the perceptions of pediatric nurses about supporting families with a child with a congenital heart defect was the view of study A9,^31^ thus creating a good relationship with the family and generating trust between all involved parties is of great importance in this context. Empowerment, as understood, provides support for structuring educational interventions developed by nurses, with practices guided by applied knowledge. Nevertheless, the understanding of this experience show that the constant changes in the health sector and the job market increasingly demand professional development, with the acquisition of knowledge, technical and relational skills, a critical-reflective stance, favoring the acquisition of skills in the developed activities.^38^

After a child who has undergone CHD surgery is discharged from hospital, care is crucial in the process of maintaining his/her health. There is a need for a way of providing care that aims at ensuring continuity of care at home. The limitations of this study are the small number of studies on the investigated topic and the limitation of texts in Portuguese, English and Spanish. It should be underlined that the search for materials for this survey identified a shortage of literature on the proposed theme, which was a limitation, since the articles found here were very old. Based on this assumption, it is clear that caring for is anchored in scientific knowledge, skill, intuition, critical thinking and creativity, as well as being accompanied by caring behaviors and attitudes. These attitudes have taken shape in nursing, giving visibility to the science it represents today.

This highlights the need for new research that seeks to underline the main nursing care and strategies for families and/or caregivers of children with congenital heart disease. Accordingly, studies such as this contribute to the awakening of a new outlook in search of the development of strategies that reach and accompany the families of children with CHD from birth, hospitalization, discharge and growth. This nursing practice corroborates the development of public policies capable of fostering the establishment of care. Nonetheless, it remains a major challenge to address this issue with such clarity and objectivity.

## Conclusion

CHD gives family members/caregivers an often negative understanding of the disease, surrounded by pain, suffering, uncertainty, doubt, loss and lack of control. Many adversities are influenced by the disease, causing caregivers to give up their lives to accompany their babies or children, meeting their demands and needs. In general, their family members show satisfaction and make a point of accompanying them throughout the process, whether it is during the discovery of the illness, through hospitalization, to discharge from hospital, not minding the fact that they have to give up their daily lives and go on with their lives according to hospital routines and treatment requirements.

There are many adversities influenced by the disease, causing caregivers to give up their lives to accompany the children and meet their demands and needs. It is assumed that the reflections made on the questions about nursing care will have a positive impact on the care provided by nurses in their scientific, technical and human spheres, given that these professionals are an integral and fundamental part of the health team, co-responsible for providing care for human beings in a holistic, integrated and individualized way.

The study showed that the nursing guidelines focus on basic care and family support for these children. Lastly, this study highlighted the important role of nurses in terms of consolidating guidelines for the care needs of these children. The articles analyzed in this research show evidence of the theme of nursing care for children with CHD, where the development of guidelines for family members and caregivers will help in a more reliable and attentive practice. In the health field, although the disease is old, there is no specific nursing guide for children with CHD, either in childhood or in adulthood. Given these aspects, it is essential that this study can help nurses and/or other health professionals to develop educational and clinical interventions in the health education field and other interventions that favor the full and correct development of care for children with CHD.
